# Production of Multiple Variants of the Antimicrobial Sactipeptide Gnavucin D by the Human Gut Isolate *Mediterraneibacter gnavus* HB038

**DOI:** 10.1002/mbo3.70315

**Published:** 2026-05-24

**Authors:** Mengfan Ding, Felipe Miceli de Farias, Paula M. O'Connor, Xiaohui Huang, Fiona C. Ross, Elaine C. Kennedy, Colin P. Hawkes, Colin Hill, Catherine Stanton, Reynolds Paul Ross

**Affiliations:** ^1^ APC Microbiome Ireland University College Cork Cork Ireland; ^2^ Teagasc Food Research Centre, Moorepark, Fermoy Cork Ireland; ^3^ Department of Pediatrics and Child Health University College Cork Cork Ireland; ^4^ INFANT Research Centre University College Cork Cork Ireland; ^5^ Perelman School of Medicine University of Pennsylvania Philadelphia Pennsylvania USA

**Keywords:** antimicrobial activity, Clostridium perfringens, gnavucin D, *Mediterraneibacter gnavus*, sactipeptide

## Abstract

While several bacteriocins have been identified from gut‐isolated cultures, there remains a need for the discovery of bacteriocins with varying inhibition spectra for strain applications such as microbiome editing, pathogen elimination and colonization resistance. With this in mind, here we describe a new antibacterial sactipeptide gnavucin D, produced by *Mediterraneibacter gnavus* HB038 isolated from a healthy 2‐year‐old child. Gnavucin D has activity against the pathogens *Clostridium perfringens*, *Streptococcus agalactiae, Bacillus cereus*, and vancomycin‐resistant *Enterococcus*. The gene cluster includes five structural genes in tandem that encode for three different core peptides (29 amino acids each) with molecular masses of 2704.21 (D1), 2734.23 (D2/D3), and 2732.21 (D4/D5) Da. The nearest relative to these bacteriocins was found to be another sactipeptide thurincin H produced by *Bacillus thuringiensis* with which it shares 30% identity. Although the amino acids encoding the gnavucin and thurincin are similar with regard to putative functions, their homology to each other is low, varying from 30% to 55%. Interestingly, all these bacteriocins had short leader peptides of only 9 amino acids. Gnavucin D was found to be extremely stable to temperature, pH and proteolysis which is possibly a reflection of the sulfur to carbon post‐translational modifications. The observed molecular masses of the 3 different peptides correspond to four modifications, yielding a structurally restricted, most likely double‐hairpin conformation which is characteristic of such sactipeptides. Consequently, gnavucin D can be a promising candidate for selective antibacterial activity against human pathogens.

## Graphical abstract

Gnavucin D, a novel antibacterial sactipeptide produced by *Mediterraneibacter gnavu*s HB038, exhibits activity against multiple pathogens, including *Clostridium perfringens*, *Streptococcus agalactiae*, *Bacillus cereus*, and vancomycin‐resistant *Enterococcus*. It contains three different peptides corresponding to four modifications, yielding a structurally restricted, most likely double‐hairpin conformation which is characteristic of such sactipeptides.

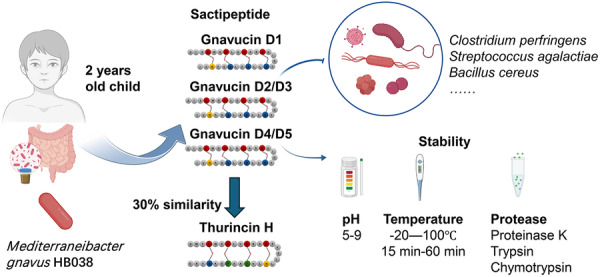

## 1 Introduction

Mammalian hosts harbor 10–100 trillion microorganisms in a predominantly symbiotic relationship (Kommineni et al. 2015). Within this microbial ecosystem, bacteriocin production is almost a ubiquitous and ecologically significant characteristic, as these ribosomally synthesized peptides are thought to mediate interspecies and intraspecies competition and participate in microbial signaling and community structure shaping (Heilbronner et al. 2021). Unlike broad‐spectrum antibiotics, bacteriocins potentially minimize disruption to the host microbiota and reduce the likelihood of resistance development, making them promising candidates for future food preservation (Mihaylova‐Garnizova et al. 2024).

Sulfur‐to‐α‐carbon (thioether) cross‐links created by the radical S‐adenosylmethionine (SAM) enzymes distinguish sactipeptides from other classes of bacteriocin (Clark, Covington, and Seyedsayamdost 2022; Rousselot‐Pailley and Iranzo 2023). Sactipeptides are compounds with a wide range of potential uses including clinical treatment, food preservation, and biological control (Ruíz‐De‐Anda et al. 2025). Some representatives of this group exhibit strong inhibitory activity against human multidrug‐resistant pathogens (Mihaylova‐Garnizova et al. 2024). To date, approximately 10 sactipeptides have been characterized, which vary in antimicrobial spectra and secretion pathways. For example, subtilosin A is released into the culture supernatant and has a moderate‐spectrum of activity against a variety of Gram‐positive and some Gram‐negative bacteria (Shelburne et al. 2006). It is a macrocyclic peptide with 35 residues, and its biosynthetic machinery is encoded by the *sbo‐alb* locus of *Bacillus subtilis* 168 (Kawulka et al. 2004). In contrast, thurincin H is a narrow‐spectrum sactipeptide that is mostly cell‐associated and produced by *Bacillus thuringiensis*. It has been demonstrated to exhibit antimicrobial action against food pathogens and phytopathogens, with its antimicrobial spectrum mostly targeting Gram‐positive bacteria (Ruíz‐De‐Anda et al. 2025). Moreover, another sactipeptide produced by *B. thuringiensis* DPC 6431 is thuricin CD, a two‐component antibacterial peptide consisting of 30‐residue sactipeptides (Rea et al. 2010). It comprises two structurally distinct components, Trnα and Trnβ, which exhibit strong synergistic activity against *Clostridioides difficile* (Rea et al. 2010).

*Mediterraneibacter gnavus* is a Gram‐positive, obligate anaerobic bacterium belonging to the family Lachnospiraceae within the phylum Firmicutes and is a prevalent member of the human gut microbiota. It is commonly detected across diverse human populations and has been reported to occur at relatively high abundance in the gastrointestinal tract. Notably, an increasing number of diseases, from inflammatory bowel disease (IBD) to metabolic disease, show positive or negative correlations with *M. gnavus* (Crost et al. 2023). These associations suggest that *M. gnavus* plays a key role in gut microbial ecology and host–microbe interactions. The excessive proliferation of *M. gnavus* in IBD patients is reported to be associated with its unique sialic acid metabolism, characterized by the release of 2,7‐dehydro‐N‐acetylneuraminic acid (2,7‐dehydro‐Neu5Ac) from host mucin (Tristancho‐Baró et al. 2024). This unique metabolic pathway may confer a competitive nutritional advantage on *M. gnavus*. Likewise, bacteriocins produced by *M. gnavus* could contribute to a competitive/dominant function. Ruminococcin C (RumC), discovered in the human gut symbiont *M. gnavus* E1 (Balty et al. 2019; Chiumento et al. 2019; Roblin et al. 2020), is non‐toxic to intestinal, colonic, or gastric cell lines, as well as human intestinal explants (Chiumento et al. 2019) (Roblin et al. 2020). Indeed, RumC1 has been reported to have beneficial effects on host cells. In a mouse model of intraperitoneal injection of clinically isolated *Clostridium perfringens*, RumC1 showed comparable efficacy to vancomycin in terms of survival, overall health status, and blood parameters (Roblin et al. 2021). These findings indicate that the bacteriocins produced by *M. gnavus* may be beneficial for the modulation of the gut microbiota.

In this study, we describe the discovery of gnavucin D, a novel sactipeptide that has antimicrobial activity against certain Gram‐positive pathogenic bacteria, including *Clostridium perfringens*, *Streptococcus agalactiae, Bacillus cereus*, and vancomycin‐resistant *Enterococcus*. Surprisingly, the gene cluster comprises five structural genes encoding three different variants.

## 2 Materials and Methods

### 2.1 Isolation and Identification of *Mediterraneibacter gnavus* HB038

*M. gnavus* HB038 was isolated from the feces of a healthy child enrolled in the Honeybiome study (Kennedy et al. 2025), which aims to characterize the gut microbiota composition of individuals under 18 years old with newly diagnosed type 1 diabetes compared to sibling controls. *M. gnavus* HB038 was isolated from a healthy sibling control. The clinicaltrials.gov registration number for the Honeybiome trial is NCT06157736.

The fecal sample was treated with a Gut Alive anaerobic stool collection kit (Microviable Therapeutics SL, Asturias, Spain) to ensure the preservation of anaerobic bacteria within the sample. For strain isolation, 100 mg of fecal sample was resuspended in 1 mL of phosphate‐buffered saline (PBS, Merck, Darmstadt, Germany) supplemented with 0.05% L‐cysteine hydrochloride (Merck) to maintain anaerobic conditions. The suspension was serially diluted and plated on five different culture media including tryptic soy broth (TSB, Millipore, Darmstadt, Germany), DeMan, Rogosa and Sharpe (MRS, Thermo Fisher Scientific Inc., Waltham, MA, USA), modified Brain Heart Infusion (MBHI) (comprising BHI (Oxoid Ltd., Basingstoke, UK) supplemented with 0.05% (w/v) L‐cysteine hydrochloride and 0.5% (w/v) yeast extract (Thermo Fisher Scientific Inc.), Glucose Minimal Medium (GMM, Thermo Fisher Scientific Inc.), and M17 medium (Thermo Fisher Scientific Inc.) in the preliminary isolation stage to maximize the recovery of diverse bacteria from the complex fecal microbiota. Plates were incubated anaerobically at 37°C for 48–72 h using a modular anaerobic workstation (Don Whitley Scientific, Bingley, UK; supplied by Davidson and Hardy, Belfast, UK). Single colonies were picked and re‐streaked twice to ensure purity, then cultured in the corresponding media. Following anaerobic incubation, pure cultures were grown overnight in MBHI broth for all subsequent experiments, then stored in 30% glycerol (Thermo Fisher Scientific Inc) at −80°C for long‐term preservation. *M. gnavus* HB038 was isolated from TSB agar plates. MBHI was utilized for subsequent cultivation to ensure standardized growth conditions across all isolates, thereby streamlining downstream analyses.

Taxonomic identification was performed via 16S rRNA gene amplification using primers spanning variable regions V1–V9. The forward primer was 27 F 5′‐AGAGTTTGATCCTGGCTCAG‐3′ and the reverse primer was 1492 R 5′‐GGTTACCTTGTTACGACTT‐3′. PCR was performed in a total volume of 20 μL using 10 μL of BioRed Mix (Meridian Bioscience, Cincinnati, OH, USA), 8 μL of nucleic acid‐free water (Thermo Fisher Scientific Inc.), 0.5 μL of forward and reverse primer (10 μM), respectively, and 1 μL of DNA template. Thermal cycling conditions were as follows: initial denaturation at 95°C for 7 min, followed by 35 cycles of 95°C for 10 s, 50°C for 30 s, 72°C for 10 s with a final extension step at 72°C for 10 min. The resulting PCR products were sent for Sanger sequencing (Azenta Life Sciences, Leipzig, Germany), and the sequences were analyzed using BLASTn against the NCBI database. The isolate was confirmed as *M. gnavus* and was designated as *M. gnavus* HB038 according to the internal biobank coding system.

The Gram staining and catalase test were employed to provide added confirmation of the identification of *M. gnavus* (Coico 2006). Briefly, A bacterial colony was prepared on a clean glass slide and heat fixed. The slide was sequentially stained with crystal violet for 1 min, iodine solution for 1 min, followed by decolorization with alcohol or acetone for 10–20 s, and then counterstained with safranin for 1 min. After each staining step, the slide was thoroughly rinsed with water. The stained slide was observed under a microscope, and the bacterial characteristics were determined based on their color reaction: purple indicates Gram‐positive, and pink indicates Gram‐negative. For the catalase test, 1 mL of 3% hydrogen peroxide (H₂O₂) was added to a sterile Eppendorf tube. A small amount of bacterial colony was picked from each strain and added to the hydrogen peroxide solution. The tube was then observed for the presence of bubbles. The presence of bubbles indicated a positive catalase reaction, confirming the presence of catalase enzyme activity in the strain. The absence of bubbles indicates a negative result, suggesting no catalase activity. The results showed *M. gnavus* is Gram positive and catalase negative (Figure S1).

### 2.2 Bacterial Strains and Culture Conditions

*M. gnavus* HB038 was routinely cultured under anaerobic conditions at 37°C in MBHI. Indicator strains, along with their respective media and growth conditions, are listed in Table 1. The media used included MBHI (Oxoid Ltd.) and M17, with the latter supplemented with 0.5% (w/v) glucose (GM17; Merck). Agar (Merck) was added at 1.5% (w/v) to prepare solid media for strain isolation or general culturing. For well diffusion assays (WDA), agar was incorporated at 0.75% (w/v) to facilitate the diffusion of test suspensions through the medium.

**Table 1:** Growth conditions of the indicator strains and inhibitory spectrum of gnavucin D in the spot‐on‐lawn assay.

Strains	Strain number	Growth temperature	Growth atmosphere	Growth media	Activity
*Clostridium perfringens*	EM124	37	Anaerobic	BHI	+++
*Streptococcus agalactiae*	35	37	Aerobic	BHI	+
*Streptococcus equi*	DSM 20561	37	Aerobic	BHI	+
*Streptococcus oralis*	UCC	37	Aerobic	BHI	+
*Bacillus cereus*	DPC 6078	37	Aerobic	BHI	++
*Mediterraneibacter gnavus*	MSB9‐43	37	Aerobic	BHI	+++
*Mediterraneibacter gnavus*	MSB9‐17	37	Aerobic	BHI	+++
*Lactococcus lactis*	HP	30	Aerobic	GM17	+++
*Escherichia coli*	ETEC	37	Aerobic	BHI	—
*Klebsiella oxytoca*	UCC	37	Aerobic	BHI	—
*Streptococcus mutans*	UCC	37	Aerobic	BHI	—
*Listeria monocytogenes*	EDGe	37	Aerobic	BHI	—
*Listeria innocua*	UCC	37	Aerobic	BHI	—
*Lactobacillus bulgaricus*	LMG 6901	37	Aerobic	BHI	—
*Staphylococcus aureus*	C5M	37	Aerobic	BHI	—
*Staphylococcus warneri*	APC 4248	37	Aerobic	BHI	—
*Staphylococcus equorum*	MA‐A‐1.a	37	Aerobic	BHI	—
*Staphylococcus epidermidis*	DSM 3095	37	Aerobic	BHI	—
VRE	APC 1033	37	Aerobic	BHI	+
VRE	APC 1038	37	Aerobic	BHI	—
VRE	APC 1028	37	Aerobic	BHI	—
VRE	APC 1037	37	Aerobic	BHI	—
VRE	APC 1039	37	Aerobic	BHI	—
VRE	APC 1031	37	Aerobic	BHI	—
VRE	APC 1029	37	Aerobic	BHI	—

Abbreviations: a, BHI, Brain‐heart infusion; GM17, glucose M17; ‐, no activity; VRE, vancomycin‐resistant enterococci; +, 0.5–5 mm inhibition zone; ++ > 5− ≤ 10 mm inhibition zone; +++ > 10 mm inhibition zone.

### 2.3 Antimicrobial Activity Assay

The antimicrobial activity of *M. gnavus* HB038 was assessed using a modified agar spot assay, based on the method described by Miceli de Farias et al. (Miceli de Farias et al. (2024) with slight modifications. Briefly, 3 μL of an overnight culture of the producer strain was spotted onto the surface of MBHI plate (Oxoid Ltd.) and incubated anaerobically at 37°C overnight. The spotted cells were then inactivated by exposure to ultraviolet light for 30 min. Subsequently, the plate was overlaid with 3 mL of soft agar (0.75% w/v, Merck) containing the indicator strain. Antimicrobial activity was assessed after further incubation at 37°C overnight, based on the presence of inhibition zones. All assays were performed in three technical replicates to ensure measurement precision and reproducibility.

### 2.4 Preparation of Cell‐Free Supernatant and Cell Extract

A 1 L culture of *M. gnavus* HB038 was grown anaerobically for 48 h at 37°C in MBHI broth, and the culture was centrifuged at 8000 × *g* for 20 min at 4°C to separate the cell pellet and supernatant (Thermo Fisher Scientific Inc.). The resulting supernatant, referred to as the cell‐free supernatant, was collected and stored on ice. The cell pellet was resuspended in 50 mL of 70% isopropanol (Merck) containing 0.1% (v/v) trifluoroacetic acid (TFA; Merck) and incubated at room temperature with shaking on a shaker (Thermo Fisher Scientific Inc.) for 3–4 h. The suspension was then centrifuged at 4000 × *g* for 10 min at 4°C and the supernatant was collected as the cell extract (Thermo Fisher Scientific Inc.). The antimicrobial activities of both the cell‐free supernatant and cell extract were evaluated using the WDA against selected indicator strains as described in Twomey et al. (2021) (Twomey et al. 2021).

### 2.5 Assessment of Heat, pH, and Protease Stability of Gnavucin D

The heat stability and pH sensitivity of gnavucin D were evaluated using WDA on the cell extracts. Samples were incubated at various temperatures (−20°C, 4°C, 37°C, 65°C, 90°C, and 100°C) for 15, 30, and 60 min using a QBD1 Digital Dry Block Heater (Grant Instruments (Cambridge) Ltd, Royston, UK). For pH stability, the cell extracts were adjusted to pH values ranging from 1.0 to 10.0 using 1 M HCl (Merck) or NaOH (Merck) and incubated at room temperature for 4 h. The pH was neutralized to 7.0 after incubation. Cell extracts of *M. gnavus* HB038 were tested, and 70% isopropanol (Merck) containing 0.1% trifluoroacetic acid (TFA; Merck) was used as a negative control and showed no zones.

To assess protease sensitivity, cell extracts were dried and resuspended in 50 mM Tris‐HCl buffer (pH 8.0, Merck). 1 mg trypsin (Sigma‐Aldrich, St. Louis, USA), chymotrypsin (Sigma‐Aldrich), and proteinase K (Sigma‐Aldrich) were dissolved 1 mL nucleic acid‐free water separately to reach a concentration of 1 mg/mL. Then 100 μL of each enzyme was added to 900 μL cell extracts in Tris‐HCl buffer at a final concentration of 100 μg/mL and incubated at 37°C for 3 h, as described by O'Connor et al (O'Connor et al. (2024). Cell extracts without enzyme treatment were tested and cell extract in Tris‐HCl buffer (pH 8.0) was used as a negative control for all the stability tests. Antimicrobial activity post‐treatment was determined via WDA on 1.5% MBHI agar and seeded with 0.5% *Clostridium perfringens* EM124 as an indicator which was isolated from fecal samples of elderly Irish subjects (Lakshminarayanan et al. 2013).

### 2.6 Genome Sequencing and Bioinformatic Analysis

Genomic DNA extraction and whole‐genome sequencing of *M. gnavus* HB038 were performed by MicrobesNG (https://microbesng.com/, MicrobesNG, Birmingham, UK) with obtaining high‐quality long reads. Sactipeptide biosynthetic gene clusters were predicted using BAGEL4 (http://bagel.molgenrug.nl) (van Heel et al. 2018) and antiSMASH v8.0 (https://antismash.secondarymetabolites.org) (Blin et al. 2023). The theoretical molecular masses of putative peptides were calculated using ProtParam (https://web.expasy.org/protparam/) (Duvaud et al. 2021). To examine the sequence similarity of putative novel bacteriocins, predicted leader and core peptide sequences were aligned with known bacteriocins and visualized using Jalview (Waterhouse et al. 2009). The alignment and similarity assessment of the amino acid sequences were calculated using Clinker (https://cagecat.bioinformatics.nl/) (Gilchrist and Chooi 2021).

### 2.7 Peptide Purification by C18 SPE and RP‐HPLC

Cell extracts of *M. gnavus* HB038 were prepared by harvesting cells via centrifugation (8000 × *g*, 15 min, 4°C) and performing acidic/methanol extraction as previously described. To prepare the sample for purification, 35 mL of the resulting cell extract was diluted with 55 mL of Milli‐Q water (Merck) to reduce the organic solvent concentration. This solution was loaded onto a 500 mg, 6 mL Strata C18‐E solid‐phase extraction (SPE) cartridge (Phenomenex, Torrance, CA, USA) that had been pre‐equilibrated with 10 mL of methanol (Merck) followed by 10 mL of Milli‐Q water (Merck). The cartridge was washed with 10 mL of 30% (v/v) ethanol (Merck) to remove hydrophilic impurities. The target peptides were subsequently eluted with 3 mL of 100% methanol (Merck). For further purification, 100 µL of the methanol eluate was mixed with 50 µL of Milli‐Q water (Merck) to match the initial mobile phase conditions and injected into an Agilent 1260 Infinity II HPLC system (Agilent Technologies, Santa Clara, CA, USA). Separation was performed on a Jupiter C18 analytical column (5 µm, 300 Å, 250 × 4.6 mm; Phenomenex) at a constant flow rate of 1.0 mL/min. The mobile phases consisted of 0.1% (v/v) trifluoroacetic acid (TFA; Merck) in water (Solvent A) and 0.1% (v/v) TFA in acetonitrile (Merck) (Solvent B). A linear gradient of 50%–65% Solvent B over 45 min was applied. Peptide elution was monitored by UV absorbance at 214 nm and 280 nm, and fractions were manually collected for subsequent antimicrobial activity assays.

## 3 Results

### 3.1 Antimicrobial Activity of *M. gnavus* HB038

Initially, we aimed to isolate human intestinal strains which would exhibit significant antimicrobial activity against human pathogenic bacteria. To do this, we screened a range of stool samples from healthy individuals in the Honeybiome study for anaerobic microorganisms inhibiting *Clostridium perfringens* as evidenced by zones of inhibition on plates overlaid with the pathogen.

Following initial identification, the antimicrobial activity of strain *M. gnavus* HB038 was further assessed using a spot‐on‐lawn assay against a wider range of bacteria. The results demonstrated an inhibitory spectrum with 9 out of 25 tested indicator strains showing sensitivity to the producer strain (Table 1). Strains exhibiting inhibition were marked with a “+” 0.5–5 mm inhibition zone; “++” > 5− ≤ 10 mm inhibition zone; “+++” > 10 mm inhibition zone. *B. cereus*, *M. gnavus*, *L. lactis*, *S. agalactiae*, *S. equi*, *S. oralis*, *C. perfringens* and one strain of vancomycin‐resistant *Enterococcus* (VRE) were inhibited by *M. gnavus* HB038.

### 3.2 Stability of Gnavucin D Under Various Conditions

The stability of gnavucin D under different temperatures, pH levels, and protease treatments was evaluated using WDA. Gnavucin D retained antimicrobial activity after incubation at –20°C, 4°C, 37°C, 65°C, 90°C, and 100°C for 15, 30, and 60 min (Table 2), indicating high thermal stability. In contrast, gnavucin D lost activity at extreme pH values (pH 1.0–4.0 and pH 10.0) but remained active between pH 5.0 and 9.0 (Table 3). Protease treatment with proteinase K, trypsin, and chymotrypsin did not affect antimicrobial activity, suggesting that gnavucin D is protease‐resistant (Table 4). The negative controls had no antimicrobial activity (Tables 2, 3, 4).

**Table 2:** Heat stability after treatment.

Temperature (°C)	15 min	30 min	60 min
Control	+	+	+
−20	+	+	+
4	+	+	+
37	+	+	+
65	+	+	+
90	+	+	+
100	+	+	+
Negative control	—	—	—

**Table 3:** pH stability after treatment.

pH	Activity
Control	+
1.0	—
2.0	—
3.0	—
4.0	—
5.0	+
6.0	+
7.0	+
8.0	+
9.0	+
10.0	—
Negative control	—

**Table 4:** Protease stability after treatment.

Treatment (100 μg/mL)	activity
Control	+
Proteinase K	+
Trypsin	+
Chymotrypsin	+
Negative control	—

### 3.3 In Silico Identification of a Bacteriocin Gene Cluster in *M. gnavus* HB038

**Figure 1:**
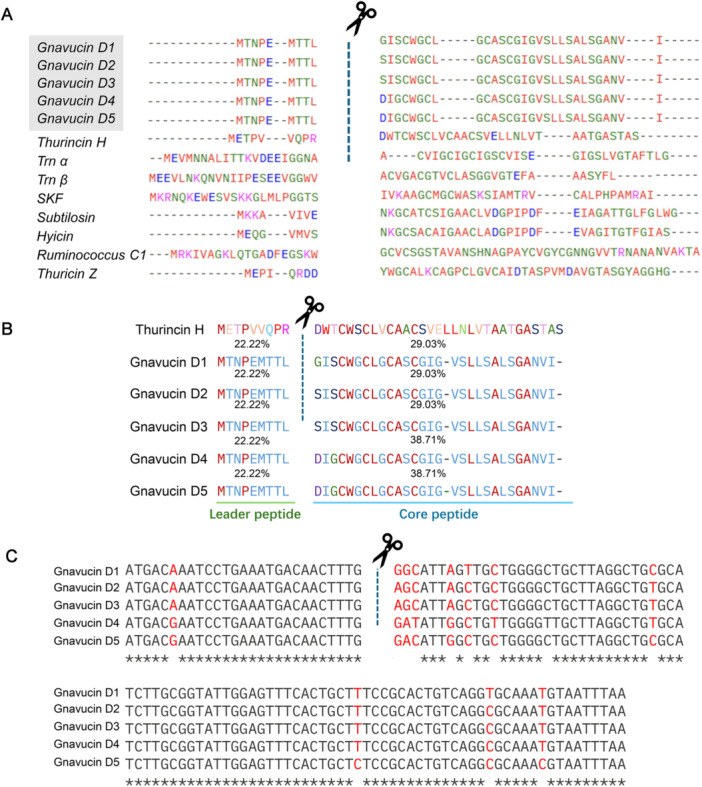
(A) Amino acid alignment of mature sactipeptides. (B) Alignment of the precursor peptides of gnavucin D and thurincin H, (C) Alignment of the nucleic acids of gnavucin D. The leader sequences are on the left side of the cleavage.

The genome sequence of *M. gnavus* HB038 was analyzed using BAGEL4 and antiSMASH v8.0 to identify potential bacteriocin gene clusters. No plasmids were identified in the genome. An approximately 10 kb chromosomal region was identified as a putative sactipeptide biosynthetic gene cluster, which showed similarity to the known sactipeptide thurincin H (Ortiz‐Rodríguez et al. 2023). This cluster is predicted to encode a novel bacteriocin, here designated gnavucin D, which comprises five structural genes (*ganD1* to *ganD5*), encoding three different core peptides presumably due to gene amplification and mutation (D2 and D3; D4 and D5) (Figure 1A).

Thurincin H belongs to sactipeptide subfamily I, characterized by the clustering of cysteine residues at the N‐terminal (left) side of the peptide (Lee et al. 2009). A multiple sequence alignment was performed including seven sactipeptides from subfamily I, as well as ruminococcin C—a sactipeptide also produced by a strain of *M. gnavus* (Figure 1 A). Despite low overall amino acid homology (30%) with thurincin H, conserved features were observed: the C‐terminal residue is frequently isoleucine, serine, glycine, or leucine. Based on alignment with thurincin H, the predicted cleavage site of gnavucin D was identified. Gnavucin D consists of a 9‐amino‐acid leader peptide followed by a 29‐amino‐acid core peptide. The leader peptide shares only 22.22% sequence identity with that of thurincin H (Figure 1B), while the core peptide exhibits 29.03% similarity to thurincin H in gnavucin D1–D3% and 38.71% in gnavucin D4–D5.

All of the gnavucin D variants have an identical leader peptide, but core peptide sequence similarity is different: D1 to D2/D3 shares 96.55% identity; D1 to D4/D5 and D2/D3 to D4/D5 both share 93.10% identity (Figure 1B), suggesting conserved functional domains among these peptides.

**Figure 2:**
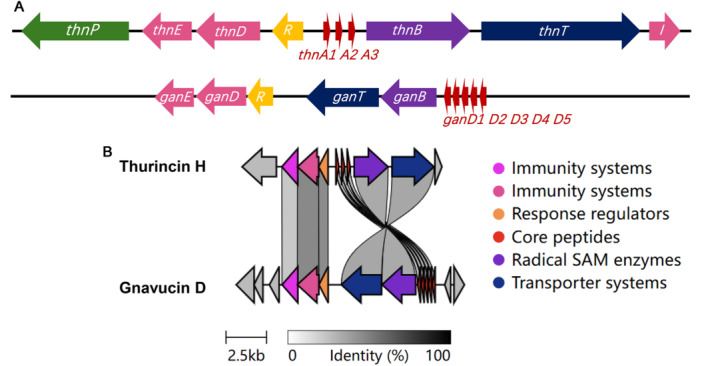
(A) The proposed gene cluster of gnavucin D (below) is based on a comparison with thurincin H (above). Purple, radical SAM enzymes; Red, precursor peptides; dark blue, transporter systems; green, signal peptidases; yellow, response regulators; pink, immunity systems. (B) The similarity of gene clusters between gnavucin D and thurincin H. The intensity of gray shading reflects the degree of similarity between gene clusters, with darker shades indicating greater similarity. The function of the genes was bioinformatically predicted based on sequence similarity to known genes in other sactibiotic gene clusters.

In silico analysis of the gnavucin D biosynthetic gene cluster revealed six key genes involved in its production: *ganE*, *ganD*, *ganR*, *ganT*, *ganB*, and *ganD1‐5* (Figure 2). The genes *ganE* and *ganD* are predicted to encode immunity proteins (32% and 55% similarity to *thnE* and *thnD*, respectively), while *ganR* encodes a response regulator potentially involved in transcriptional regulation (54% similarity to *thnR*). The gene g*anT* encodes an ABC transporter (30% similarity to *thnT*), likely responsible for peptide export. Notably, *ganB* encodes a radical SAM enzyme (30% similarity to *thnR*), a hallmark of sactipeptide biosynthesis, catalyzing the formation of thioether cross‐links. The *ganD1‐5* genes encode the core peptides of gnavucin D.

Compared to the well‐characterized thurincin H gene cluster, the gnavucin D cluster is more compact, with fewer associated genes. Notably, genes encoding leader peptide‐processing peptidases were not identified in the gnavucin D cluster, while thurincin H includes specific genes for this function. Moreover, thurincin H possesses three distinct immunity‐related genes, whereas only two were detected in gnavucin D (Figure 2 A). Despite these differences, several genes within the gnavucin D cluster show sequence similarity to those in the thurincin H cluster, suggesting shared biosynthetic features (Figure 2B).

### 3.4 Bacteriocin Purification

**Figure 3:**
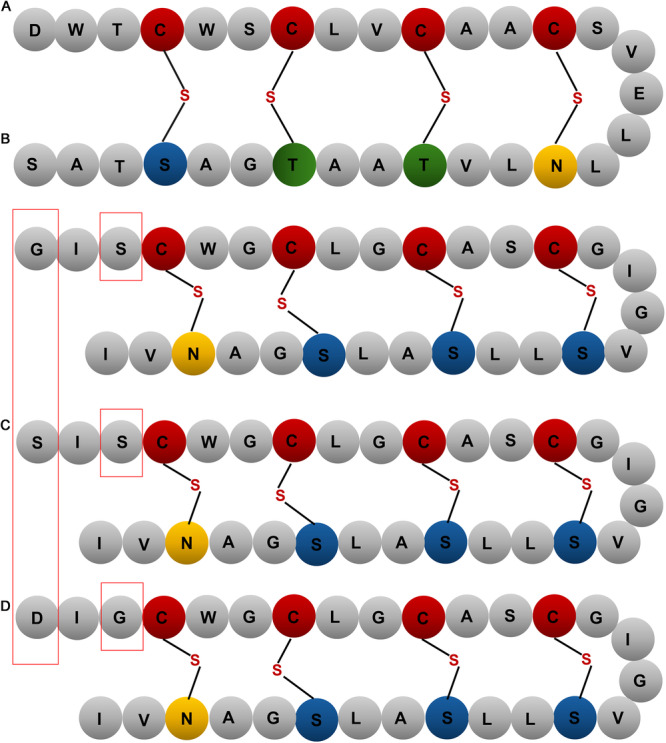
The predicted structure of gnavucin D based on the structure of thurincin H. (A) Thurincin H. (B) Gnavucin D1. (C) Gnavucin D2/D3. (D) Gnavucin D4/D5. Amino acids in red boxes highlight the variation among gnavucin core peptides. The structure of gnavucin D1‐5 was predicted based on the structure of thurincin H and would need to be confirmed by structural determination.

The molecular mass of gnavucin D was initially predicted based on the amino acid sequences of its mature peptides yielding theoretical masses of 2712.21 Da (D1), 2742.23 Da (D2/D3) and 2740.22 Da (D4/D5). As a member of the sactipeptide family, gnavucin D is expected to contain sulfur‐to‐α‐carbon thioether cross‐links, a hallmark of this class of bacteriocins, formed by radical SAM enzymes. Each thioether bridge results in a 2 Da mass loss in molecular mass due to the elimination of two hydrogen atoms during bond formation. Based on structural comparisons with thurincin H, and molecular mass determinations we would predict that gnavucin D has 4 cross bridges. Accordingly, the adjusted molecular masses are estimated to be 2704.21 Da (D1), 2734.23 Da (D2/D3) and 2732.22 Da (D4/D5) (Figure 3).

**Figure 4:**
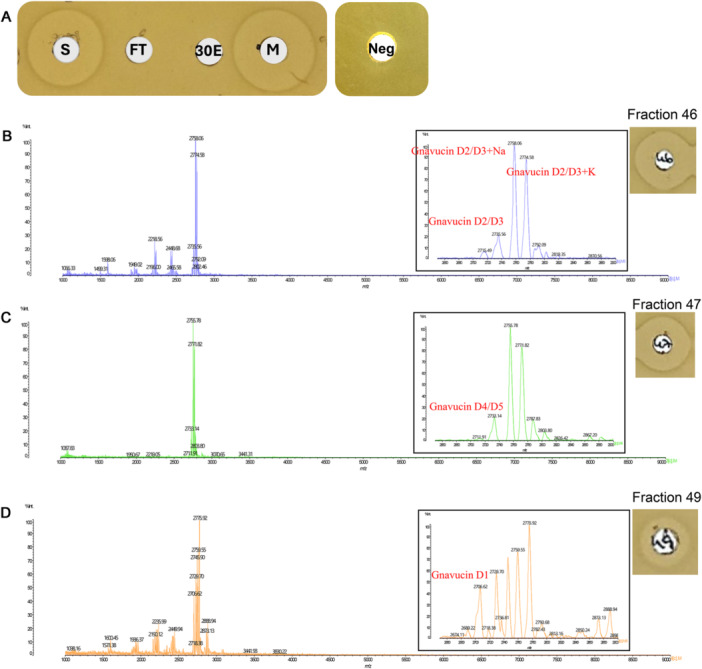
The predicted molecular mass of gnavucin D by MALDI‐TOF mass spectrum. (A) Activity test of C18 SPE eluents. (B) Detection of the mass of gnavucin D2/D3 and gnavucin D2/D3 with adducts (Na^+^/K^+^). (C) Detection of the mass of gnavucin D4/D5. (D) Detection of the mass of gnavucin D1.

Initially, cell extracts and cell‐free supernatants were evaluated for antimicrobial activity using WDA. As bioactivity was observed only in the cell extract it was selected for further purification. It was applied to a C18 SPE column, and the eluted fractions were tested for activity using *Lactococcus lactis* HP as an indicator strain. Antimicrobial activity (S) was retained by the C18 SPE column as neither the column flow‐through (FT) nor the 30% ethanol wash showed inhibition (30E) while activity was eluted in the methanol eluate (M) confirming that the active compound was retained and subsequently eluted from the C18 column. Methanol alone (Neg) showed no inhibitory effect on *L. lactis* HP (Figure 4A), indicating that the observed activity was attributable to gnavucin D. The methanol eluate was further purified by reversed‐phase HPLC, and active fractions were subjected to matrix‐assisted laser desorption/ionization time‐of‐flight (MALDI‐TOF) mass spectrometry to assess gnavucin masses. Analysis of fractions 45‐49 revealed major peaks at 2774.58, 2758.06, 2735.56 2733.14, and 2706.62 Da. The 2706.62 Da peak closely matches the predicted mass of the gnavucin D1 peptide (2704.12 Da ± 2 Da). The peaks at 2735.56 and 2733.14 closely correspond to the calculated molecular masses of gnavucin D2/D3 (2734.23 Da) and gnavucin D4/D5 (2732.22 Da), respectively. The peaks at 2758.06 and 2774.58 Da correspond to sodium‐ and potassium‐adduct forms of the D2/D3 variants (theoretical mass 2734.23 Da + 22 Da and + 38 Da, respectively, Figure 4B). The similar molecular masses of these three variants probably explain why it was difficult to separate them individually during purification/concentration.

## 4 Discussion

A novel sactipeptide, referred to herein as gnavucin D was identified in this study. It is produced by *M. gnavus* isolated from the fecal sample of a 2‐year‐old child. The gene cluster for gnavucin D includes five structural genes that encode three variants. Genomic analysis revealed predicted molecular masses of 2704.21 Da (D1), 2734.23 Da (D2/D3), and 2732.22 Da (D4/D5) and purification from a cell extract confirmed the presence of these masses in HPLC fractions that displayed antimicrobial activity. Gnavucin D retained its antimicrobial activity after exposure to pH values ranging from 5.0 to 9.0, heat treatment up to 100°C for 60 min and treatment by proteinase K, trypsin and chymotrypsin.

Through spot‐on‐lawn testing, we discovered that gnavucin D inhibits nine of the twenty‐five tested bacterial strains. The compound demonstrates selective antibacterial activity across five distinct bacterial genera. While the most potent effect was observed against *Clostridium perfringens*, the broad taxonomic diversity of the susceptible strains suggests a targeted rather than restricted host range. Gnavucin D is a sactipeptide that inhibits the growth of Gram‐positive bacteria including *B. cereus*, *M. gnavus*, *L. lactis*, *S. agalactiae*, *S. equi*, *S. oralis*, *C. perfringens*, and one strain of vancomycin‐resistant *Enterococcus* (VRE). This selective behavior indicates that gnavucin D may target conserved cellular components seen in distinct phylogenetic groupings. Interestingly, different sactipeptides act on different targets. For example, thuricin CD, produced by *B. thuringiensis* DPC 6431, exhibits highly selective activity, primarily targeting *C. difficile* (Mathur 2014) while subtilosin A exhibits antimicrobial activity against selected Gram‐positive organisms, including *Listeria monocytogenes, Gardnerella vaginalis* and *S. agalactiae* (Amrouche et al. 2010). This demonstrates that different bacteriocins can be selected for different pathogens to achieve targeted inhibitory effects. Furthermore, the ability of gnavucin D to inhibit therapeutically relevant bacteria such as *C. perfringens* and vancomycin‐resistant *Enterococcus* demonstrates its potential as a targeted antimicrobial agent, particularly in the treatment of multidrug‐resistant pathogens (van Harten et al. 2017).

In addition to its antimicrobial activity, gnavucin D demonstrated remarkable physicochemical stability under various stress conditions. While thuricin CD has been reported to be heat‐stable up to approximately 90°C for 15 min (Rea et al. 2010), gnavucin D retained activity even after exposure to 100°C for up to 60 min. Such thermostability is a desirable feature for potential industrial applications, where temperature fluctuations are common during processing or storage. Furthermore, gnavucin D retained its antibacterial activity over a relatively broad pH range (pH 5.0–9.0), while activity was lost under more extreme acidic (pH 1.0–4.0) and alkaline (pH 10.0) conditions. However, thuricin CD demonstrates high pH stability, retaining bioactivity between pH 2.0 and 9.0 (Hill et al. 2014; Rea et al. 2010) which shows broader pH stability, especially towards lower acidic conditions. These results indicate that gnavucin D exhibits weaker pH tolerance compared to thuricin CD. Therefore, the selection of an appropriate sactipeptide can be guided by its specific physicochemical properties for practical applications.

Sactipeptides exhibit considerable variability in mass when compared to the theoretical mass of the structural peptides due to their complex cross‐linked structures and post‐translational modifications (Ongpipattanakul et al. 2022). This mass difference helps to deduce the likely post‐translational modifications present in the sactipeptide. Gnavucin D peptides were purified from a cell extract using a 2‐step purification protocol that included C18 SPE and reversed‐phase HPLC. The molecular masses of gnavucin D structural peptides are 2712.21 Da (D1), 2742.23 Da (D2/D3) and 2740.22 Da (D4/D5) as calculated based on their amino acid sequence. The observed masses (2704.21 Da (D1), 2734.23 Da (D2/D3), and 2732.22 Da (D4/D5)) differed from the theoretical values calculated based on the modified amino acid sequences by 8 Da which could potentially be attributed to the presence of four thioether cross‐links. These minor differences suggested the presence of post‐translational modifications, which is consistent with the known structural complexity of sactipeptides that often exhibit characteristic mass shifts due to thioether cross‐links. In addition, the molecular masses of gnavucin D observed in MALDI‐TOF MS, such as 2758.06 Da and 2774.58 Da, may indicate the presence of additional modifications, possibly due to the binding of metal ions such as Na^+^ or K^+^ (Laval et al. 2001). This is noteworthy, as these cations often play a role in the structural stabilization or membrane‐targeting efficiency of bacteriocins. This binding capability might further influence its activity under different physiological environments.

Gnavucin D exhibits structural stability comparable to thurincin H, which features four thioether bridges conferring resistance to proteolysis and heat, as demonstrated by its own remarkable enzymatic and thermal stability. However, unlike several other sactipeptides released into the culture supernatant (Algburi et al. 2017; Ortiz‐Rodríguez et al. 2023), such as subtilosin A and thuricin CD, gnavucin D appears to be cell‐associated. The cell‐associated localization of gnavucin D not only distinguishes it from other sactipeptides, possibly reflecting differences in secretion machinery or membrane affinity. This trait may impact both its biological function and how to purify it, setting it apart from other widely distributed sactipeptides such as thurincin H and subtilosin A. Furthermore, the predicted gnavucin D transporter is shorter than its thurincin H homolog. Current research has revealed that some bacteriocin biosynthetic gene clusters lack all the genes required for biosynthesis, often relying on hidden enzymes encoded elsewhere in the genome (Xue et al. 2022). For example, the lanthipeptide ‐thiopeptide biosynthetic gene clusters typically do not contain protease genes, such as paenilan, and its maturation requires the external protease Prot_686, located approximately 550 kb away (Xue et al. 2022). Some bacteriocins, such as enterococcin F4‐9, undergo a two‐step process: initial cleavage by a transporter followed by final maturation by a specific host‐produced extracellular protease, also located outside the biosynthetic gene clusters (Maky et al. 2021). In some systems, the processing steps may involve multiple functional domains of an enzyme or a conserved protease system located outside the biosynthetic gene clusters. Therefore, the peptidase responsible for processing the gnavucin D leader peptide could be encoded in another genomic region.

The mechanism of action of sactipeptides has been a focus of diverse studies but remains not well understood. Some type I sactipeptides present a lytic mode of action (thuricin CD, subtilosin A, thuricin Z/huazacin) and others a non‐lytic mode of action (thurincin H). Mathur and colleagues (2017) suggested that the lytic activity of thuricin CD involved its insertion into the membrane of the cells leading to membrane depolarization and cell death due to pore formation (Mathur et al. 2017). For thuricin Z/huazacin, a combination of microscopy and large‐unilamellar‐vesicle‐based fluorescence assays supports the hypothesis that the bactericidal activity of the peptide is also caused by membrane permeabilization (Mo et al. 2019). Regarding subtilosin A, the most well‐studied peptide of the group, a study revealed that the bacteriocin formed transient pores leading to loss of intracellular ions and ATP, which resulted in the death of *G. vaginalis* (Sutyak Noll et al. 2011). In contrast, thurincin H does not demonstrate lytic behavior. A study revealed that the peptide, even when used at 32X the minimal inhibitory concentration, does not alter the rod morphology of the sensitive strain *B. cereus* F4552. Due to the absence of a drop in the optical density associated with no alteration of the membrane permeability, it was proposed that the mechanism of action does not involve pore formation (Wang et al. 2014). Due to the similarity between thurincin H and gnavucin D, it is possible that both peptides share a similar antimicrobial mode of action. Future research is needed to understand its mechanism of action, receptor selectivity, and possible synergistic effects when paired with other antimicrobials.

*M. gnavus* has potential duality in the human microbiome, switching between symbiotic and pathogenic states depending on the host environment (Juge 2023). Although present in over 90% of the population, its overgrowth is a hallmark of gut microbiota dysbiosis (Juge 2023). On the harmful side, *M. gnavus* is associated with IBD, metabolic syndrome including type 2 diabetes and Metabolic Dysfunction‐Associated Steatotic Liver Disease (MASLD) and may exacerbate hepatic steatosis and insulin resistance (Crost et al. 2023; Meadows et al. 2025). Its early colonization in some infants could mean that the organism is a predictor of respiratory allergies (Juge 2023). One of the potential beneficial aspects of *M. gnavus* lies in its bacteriocins, such as ganvucin D (discovered in this paper) and the previously reported RumC. For example, RumC can eliminate clinical pathogens such as *Clostridium difficile* and methicillin‐resistant *Staphylococcus aureus* at micromolar concentrations without damaging the integrity of human epithelial cells (Shamseddine et al. 2023). Ganvucin D effectively inhibits the growth of pathogenic bacteria in vitro, but its in vivo efficacy needs further investigation. These peptides, modified by free radical SAM enzymes, possess a unique double hairpin structure which probably contributes to their excellent heat resistance, pH stability, and resistance to digestive proteases (Shamseddine et al. 2023). Therefore, *M. gnavus* cannot be simply defined as a pathogen or symbiont.

## 5 Conclusions

In summary, we characterized gnavucin D, a novel sactipeptide from *M. gnavus* HB038 with robust stability and potent activity against Gram‐positive pathogens such as *C. perfringens*. While the discovery of such a potent antimicrobial suggests significant applications for gut microbiome editing and pathogen exclusion, its origin in *M. gnavus* presents a complex dilemma with regard to exploitation. As *M. gnavus* is frequently associated with IBD and intestinal dysbiosis, the production of gnavucin D could serve as a competitive factor that facilitates the overgrowth of its producer at the expense of beneficial commensals. This dual nature implies that while gnavucin D itself holds promise as a selective antimicrobial, its ecological role in situ may contribute to the unwelcome persistence of a strain. However, the isolated bacteriocin may alternatively be used to safely harness its narrow‐spectrum activity for future use.

## Author Contributions

**Mengfan Ding:** conceptualization, writing – original draft. **Felipe Miceli De Farias:** methodology, investigation. **Paula M. O'Connor:** methodology. **Xiaohui Huang:** methodology, investigation. **Fiona C. Ross:** methodology. **Elaine C. Kennedy:** methodology, investigation. **Colin P. Hawkes:** methodology, investigation. **Colin Hill:** writing – review and editing. **Catherine Stanton:** writing – review and editing. **Reynolds Paul Ross:** writing – review and editing.

## Ethics Statement

The clinicaltrials.gov registration number for this trial is NCT06157736. The registration date was 23rd of February 2023.

## Consent

All participants involved in this study provided their informed consent for the publication of anonymized data.

## Conflicts of Interest

The authors declare no conflicts of interest.

## Supporting information

Supporting File:

## Data Availability

The data that support the findings of this study are available on request from the corresponding author. The data are not publicly available due to privacy or ethical restrictions.
