# Supplementary figures and images for: Production of Multiple Variants of the Antimicrobial Sactipeptide Gnavucin D by the Human Gut Isolate Mediterraneibacter gnavus HB038
Source: Microbiologyopen. 2026 May 24;15(3):e70315. doi: 10.1002/mbo3.70315 (PMC13239113; doi:10.1002/mbo3.70315)

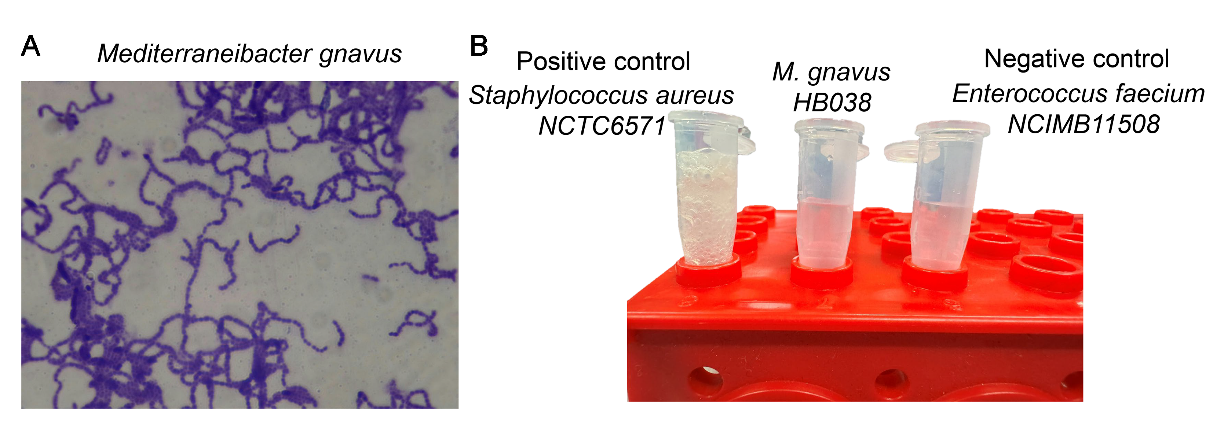


Figure S1 (A) Gram staining and (B) catalase test of *Mediterraneibacter gnavus*.

Supplement: Supplementary file 1 — Supporting File: [file MBO3-15-e70315-s001.docx]
